# Associations of different exercise modalities with balance performance and cortical hemodynamic responses in older adults with mild cognitive impairment

**DOI:** 10.3389/fphys.2026.1870532

**Published:** 2026-07-08

**Authors:** Hongen Liu, Xuecheng Zhang, Yanbai Han, Yiming Han

**Affiliations:** 1 Guangxi Normal University, Guilin, China; 2 Shandong Provincial Hospital Affiliated to Shandong First Medical University, Jinan, China

**Keywords:** balance performance, cortical hemodynamic responses, exercise, fNIRS, mild cognitive impairment

## Abstract

**Objective:**

To compare balance performance and task-related cortical hemodynamic responses among older adults with mild cognitive impairment (MCI) who habitually engaged in different exercise modalities, and to provide preliminary evidence for future balance-oriented intervention studies and fall-risk prevention strategies.

**Methods:**

This cross-sectional observational study included 57 older adults with MCI, classified into Tai Chi, brisk walking, and non-exercise control groups according to habitual exercise patterns, with 19 participants in each group. Static balance was assessed during quiet standing, and dynamic balance was evaluated using the Timed Up and Go (TUG) test and the Tinetti Performance-Oriented Mobility Assessment (POMA). Functional near-infrared spectroscopy was used to record oxygenated hemoglobin (HbO) concentrations in predefined regions of interest during quiet standing and TUG tasks.

**Results:**

Compared with the control group, both the Tai Chi and brisk walking groups showed significantly lower postural sway during quiet standing and higher POMA scores during dynamic balance assessment (*p* < 0.05). The Tai Chi group also showed significantly shorter TUG completion time than the control group (*p* < 0.05) and demonstrated more pronounced advantages across static balance outcomes than the brisk walking group (*p* < 0.05). Cortical HbO results showed that the Tai Chi group had significantly higher HbO concentrations in the left primary somatosensory cortex during quiet standing and in the right dorsolateral prefrontal cortex during walking compared with the control group (*p* < 0.05), whereas no significant differences were observed between the brisk walking and control groups.

**Conclusion:**

Different habitual exercise modalities were associated with better balance control in older adults with MCI, with Tai Chi showing more pronounced favorable associations than brisk walking. Habitual Tai Chi practice may be linked to better postural regulation and task-related cortical hemodynamic responses, supporting its potential relevance for future balance-oriented intervention studies and fall-risk prevention strategies in older adults with MCI.

## 1 Introduction

Mild cognitive impairment (MCI) refers to a clinical condition characterized by progressive decline in memory or other cognitive domains. Although such cognitive decline does not substantially interfere with independence in activities of daily living and does not meet the diagnostic criteria for dementia, individuals with MCI frequently exhibit not only impairments in core cognitive functions, such as memory, attention, and executive function, but also deterioration in motor function. This motor decline is particularly reflected in impaired balance performance and an increased risk of falls (Montero-Odasso et al., 2018). Compared with cognitively healthy older adults, older adults with cognitive impairment have a markedly higher risk of falls (Fernando et al., 2017), and this risk becomes more pronounced as cognitive impairment progresses. For example, the incidence of falls among older adults with dementia is approximately two to three times higher than that among cognitively healthy older adults, with an estimated 60–80% of patients with dementia experiencing at least one fall each year (Hopkins et al., 2023; Beauchet et al., 2019). Although fall risk is particularly pronounced in dementia, balance impairment may emerge earlier during the MCI stage, highlighting the importance of early identification and intervention. Therefore, it is important to identify safe and sustainable strategies to maintain or improve balance performance in older adults with MCI.

Exercise is widely recognized as an effective non-pharmacological intervention for attenuating cognitive decline and improving balance performance in older adults. The scaffolding theory of aging and cognition proposes that sustained behavioral engagement and accumulated experience can promote the formation of compensatory neural networks, thereby mitigating age-related cognitive deterioration (Reuter-Lorenz and Park, 2024). From physiological and neurobiological perspectives, exercise may enhance functional capacity through multiple pathways. Regular exercise not only improves cardiorespiratory fitness, muscle strength, and balance performance, but also promotes neuroplasticity during the learning and execution of motor skills, strengthens sensorimotor integration, and upregulates the expression of neurotrophic factors such as brain-derived neurotrophic factor, thereby providing a neurobiological basis for cognitive improvement (Etnier et al., 2016; Kasahara et al., 2025; Tomporowski and Pesce, 2019). Owing to its safety, low cost, and sustainability, exercise has increasingly been regarded as a promising intervention strategy for promoting cognitive health in older adults (Zhang et al., 2023; Biazus-Sehn et al., 2020; Liu et al., 2023).

With the advancement of neuroimaging techniques, increasing attention has been directed toward elucidating the central mechanisms underlying postural balance from the perspective of brain function. Previous studies have shown that maintaining posture or performing gait tasks is accompanied by significant changes in neural activity across multiple brain regions (Ding et al., 2024; Chen et al., 2022a; Feng et al., 2023). These changes involve not only motor-related areas, such as the motor cortex, cerebellum, and basal ganglia, but also cortical regions closely associated with cognitive and sensorimotor processing, including the prefrontal (Gozdas et al., 2024) and parietal cortex (Sarasso et al., 2022). The prefrontal cortex, particularly the dorsolateral prefrontal cortex, is involved in attentional allocation, executive control, motor planning, and cognitive–motor regulation during postural and walking tasks. The parietal cortex contributes to somatosensory processing, proprioceptive integration, and body-position monitoring, which are essential for maintaining balance. Functional near-infrared spectroscopy (fNIRS), as a non-invasive neuroimaging technique, enables the monitoring of cortical hemodynamic responses during movement and has therefore been increasingly applied to research on motor–cognitive interactions.

Despite growing interest in exercise-based interventions for older adults with MCI, no consensus has been reached regarding the comparative effects of different exercise modalities on balance performance in this population. Conventional aerobic exercise may improve balance performance indirectly by increasing overall physical activity levels and enhancing cardiorespiratory fitness. In contrast, Tai Chi is a multimodal mind–body exercise characterized by slow weight shifting, controlled postural transitions, trunk rotation, and coordinated upper- and lower-limb movements, which may more directly engage postural stability, sensorimotor integration, and fall-risk-related balance control (Sungkarat et al., 2017; Chen et al., 2023). However, whether Tai Chi provides greater benefits than conventional aerobic exercise in improving balance control among older adults with MCI remains unclear and requires further empirical validation.

Moreover, existing evidence has largely been derived from review-based studies, whereas empirical studies directly comparing different exercise modalities remain relatively limited. In particular, evidence is still lacking on whether different exercise modalities are associated with distinct cortical hemodynamic response patterns in older adults with MCI. Therefore, the present study adopted a cross-sectional design to compare differences among older adults with MCI engaging in different exercise modalities from both behavioral and neural regulatory perspectives. By including a Tai Chi group, a brisk walking group, and a non-exercise control group, this study aimed to examine whether different habitual exercise modalities were associated with differences in balance control and task-related cortical hemodynamic responses in older adults with MCI.

## 2 Participants and methods

### 2.1 Participants

#### 2.1.1 Sample size calculation

Because no directly comparable effect-size estimates were available for the combined behavioral and fNIRS outcomes in older adults with MCI, sample size estimation was conducted based on an exploratory large-effect assumption (Greenfield et al., 1997). Using G*Power 3.1 for one-way ANOVA with three independent groups, with f = 0.45, α = 0.05, and power = 0.80, the minimum required sample size was 51 participants. To account for potential invalid behavioral or fNIRS data, 57 participants were recruited, with 19 participants in each group.

#### 2.1.2 Eligibility criteria

According to participants’ previous exercise habits, 57 older adults with MCI were recruited from local communities, including 19 participants each in the Tai Chi group, brisk walking group, and control group.

All participants were required to meet the following inclusion criteria: (1) aged ≥ 65 years; (2) participants were diagnosed with MCI according to established clinical criteria, including subjective cognitive decline, objective cognitive impairment, largely preserved activities of daily living, and absence of dementia. Global cognitive function was screened using the Montreal Cognitive Assessment (MoCA), with scores between 20 and 26 used as an inclusion criterion (Petersen et al., 1999; Albert et al., 2011; Nasreddine et al., 2005); (3) no history of cardiovascular, respiratory, musculoskeletal, or neurological disorders, and no visual impairment affecting normal vision; (4) no use of medications affecting balance or the nervous system within the past six months; (5) ability to walk independently; and (6) right-handedness.

Additional group-specific criteria were applied. Exercise type, years of exercise experience, and weekly exercise frequency were confirmed through face-to-face interviews before enrollment. Participants in the Tai Chi group were required to have regularly practiced simplified 24-form Tai Chi for at least 12 consecutive months, with a frequency of ≥3 sessions per week and a duration of ≥45 min per session. Participants in the brisk walking group were required to have engaged in moderate-intensity brisk walking for at least 12 consecutive months, with a frequency of ≥3 sessions per week and a duration of ≥45 min per session. Moderate-intensity brisk walking was defined as walking faster than usual, inducing noticeable increases in breathing and heart rate while still allowing conversation. Participants in the control group had not participated in regular structured exercise training within the past 12 months. No significant differences in baseline characteristics were observed among the three groups (**Table 1**).

**Table 1:** Baseline characteristics of the participants.

Variable		Tai Chi group (n = 19)	Brisk walking group (n = 19)	Control group (n = 19)	*F/t/χ^2^*	*p*
Sex	Male	4	6	6	0.620	0.733
	Female	15	13	13		
Age (years)		66.72 ± 3.11	67.44 ± 3.65	67.00 ± 2.29	0.279	0.758
Height (cm)		159.58 ± 6.38	160.06 ± 6.99	163.42 ± 7.42	1.883	0.162
Weight (kg)		65.70 ± 6.77	63.57 ± 7.42	67.01 ± 10.53	0.873	0.425
BMI (kg/m^2^)		25.83 ± 2.55	24.79 ± 2.13	24.95 ± 2.25	1.110	0.336
Education level	Primary school or below	7	8	6	1.036	0.900
	Junior high school	7	8	9		
	Senior high school	5	3	4		
MoCA score		22.67 ± 1.94	23.17 ± 0.76	22.11 ± 2.13	2.728	0.074
Exercise experience (years)		3.47 ± 0.86	3.71 ± 0.89	–	-0.846	0.403
Exercise frequency (sessions/week)		4.31 ± 0.23	4.56 ± 0.26	–	-1.885	0.067
GDS-15		4.28 ± 0.80	4.06 ± 0.52	4.00 ± 0.82	1.092	0.343
PSQI		5.56 ± 0.68	5.28 ± 0.56	5.61 ± 0.68	1.517	0.228
SF-36		85.47 ± 12.76	84.51 ± 11.89	81.02 ± 9.18	0.787	0.461

MoCA, Montreal Cognitive Assessment; BMI, Body Mass Index; GDS, Geriatric Depression Scale–Short Form; PSQI, Pittsburgh Sleep Quality Index; SF-36, 36-Item Short Form Health Survey.

This study was conducted in accordance with the Declaration of Helsinki and was approved by the Ethics Committee of Shandong Sport University (No. 2024066). All participants were fully informed of the study procedures, content, and requirements, and provided written informed consent before participation.

#### 2.1.3 Bias control and blinding

Participants were classified into groups according to their long-term habitual exercise patterns; therefore, this study used a cross-sectional natural grouping design. Accordingly, random allocation and double blinding at the intervention level were not feasible. To minimize potential bias, a random number generator was used before data collection to randomize participant identification numbers and the order of testing procedures. Participants were then assessed according to the randomized sequence to reduce potential order effects and fatigue-related influences on the measurement outcomes.

All assessments were performed according to standardized protocols by uniformly trained investigators who were not involved in group classification. During data processing and statistical analysis, all raw data were anonymized after export, and group information was replaced with coded labels. The data analyst remained blinded to the participants’ group information throughout the statistical analysis, thereby minimizing potential analytical bias in the interpretation of the results.

### 2.2 Experimental design

This study adopted a cross-sectional observational controlled design with one factor and three levels: the Tai Chi group, brisk walking group, and control group. All participants completed center of pressure (COP) trajectory assessment during bipedal quiet standing, the Timed Up and Go (TUG) test, and the Tinetti Performance-Oriented Mobility Assessment (POMA).

**Figure 1:**
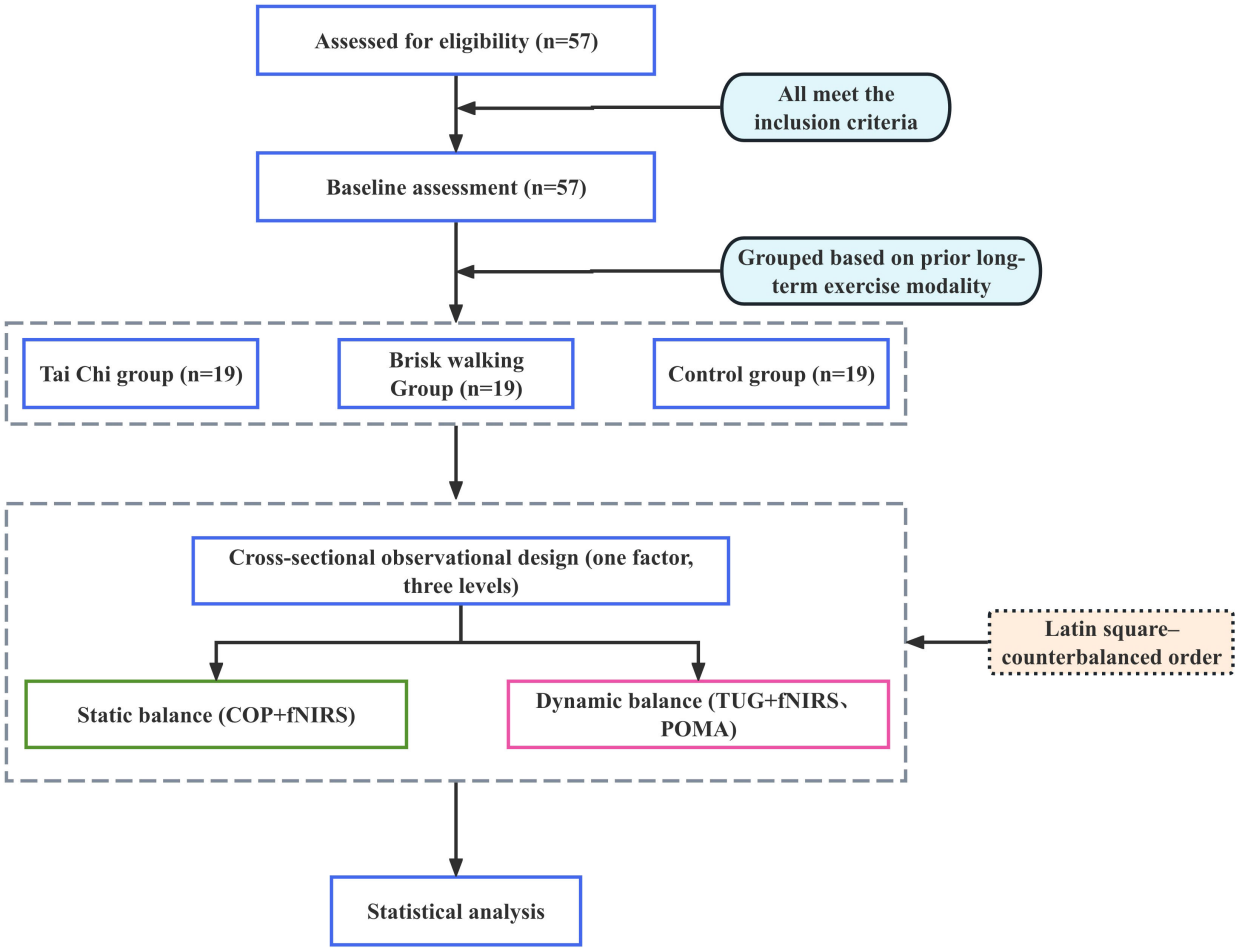
Schematic diagram of study design.

During the bipedal quiet standing and TUG tasks, fNIRS was simultaneously used to record changes in oxygenated hemoglobin (HbO) concentrations in predefined regions of interest (ROIs) (**Figure 1**).

### 2.3 Procedures and methods

#### 2.3.1 Static balance assessment

**Figure 2:**
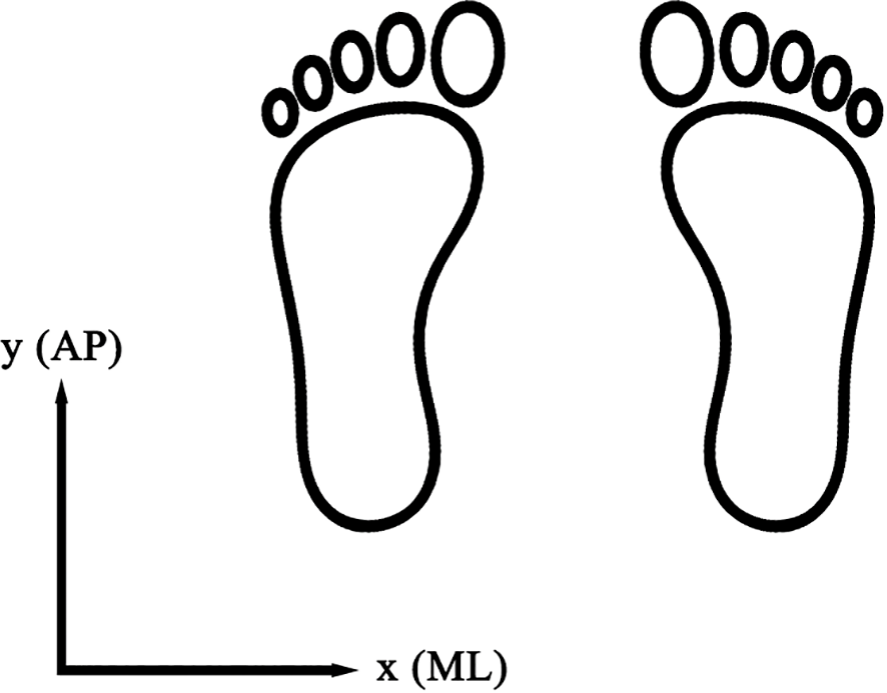
Force platform coordinate system.

Participants performed quiet standing tasks under two visual conditions: eyes-open (EO) and eyes-closed (EC) bipedal stance. The order of the EO and EC conditions was counterbalanced across participants. Each condition was repeated three times, with each trial lasting 30 s and a 1-min rest interval between trials. Before data collection, the origin of the force platform was calibrated. The medio–lateral (ML) direction was defined as the x-axis, and the anterior–posterior (AP) direction was defined as the y-axis (**Figure 2**). Participants were instructed to stand barefoot on the platform with their feet parallel and approximately 20 cm apart, while keeping their arms relaxed at their sides. In the EO condition, participants were asked to fixate on a visual reference marker positioned directly in front of them. A trial was considered invalid if either foot moved during the recording period. Ground reaction force signals were collected using a three-dimensional force platform (AMTI-BP600900, AMTI, USA; dimensions: 90 cm × 60 cm × 10 cm) at a sampling frequency of 1000 Hz.

#### 2.3.2 Dynamic balance assessment

TUG: Each participant completed three TUG trials, with a 1-min rest interval between trials. The test was conducted in a quiet, obstacle-free, level, and non-slip indoor environment. Before testing, a marker was placed 3 m from the starting position to indicate the walking distance. Participants were seated on a standard armless chair with a seat height of approximately 43–45 cm, with both feet placed naturally on the floor and both hands resting on the thighs. At the verbal command “start,” the assessor started the stopwatch. Participants were instructed to stand up, walk in a straight line to the 3-m marker at their usual walking speed, turn around the marker, walk back to the chair, and sit down again. Timing was stopped when the participant returned to the seated position. Participants were instructed to complete the task as continuously as possible. A trial was considered invalid if the participant showed a marked pause during walking or required external assistance.

POMA: The POMA was administered in a quiet and level environment by uniformly trained assessors following standardized procedures. Participants wore their usual shoes; none used assistive devices during testing. The balance and gait subscales were assessed sequentially. Each item was scored according to the participant’s actual performance, and the balance subscale score, gait subscale score, and total score were then calculated.

#### 2.3.3 fNIRS data acquisition

**Figure 3:**
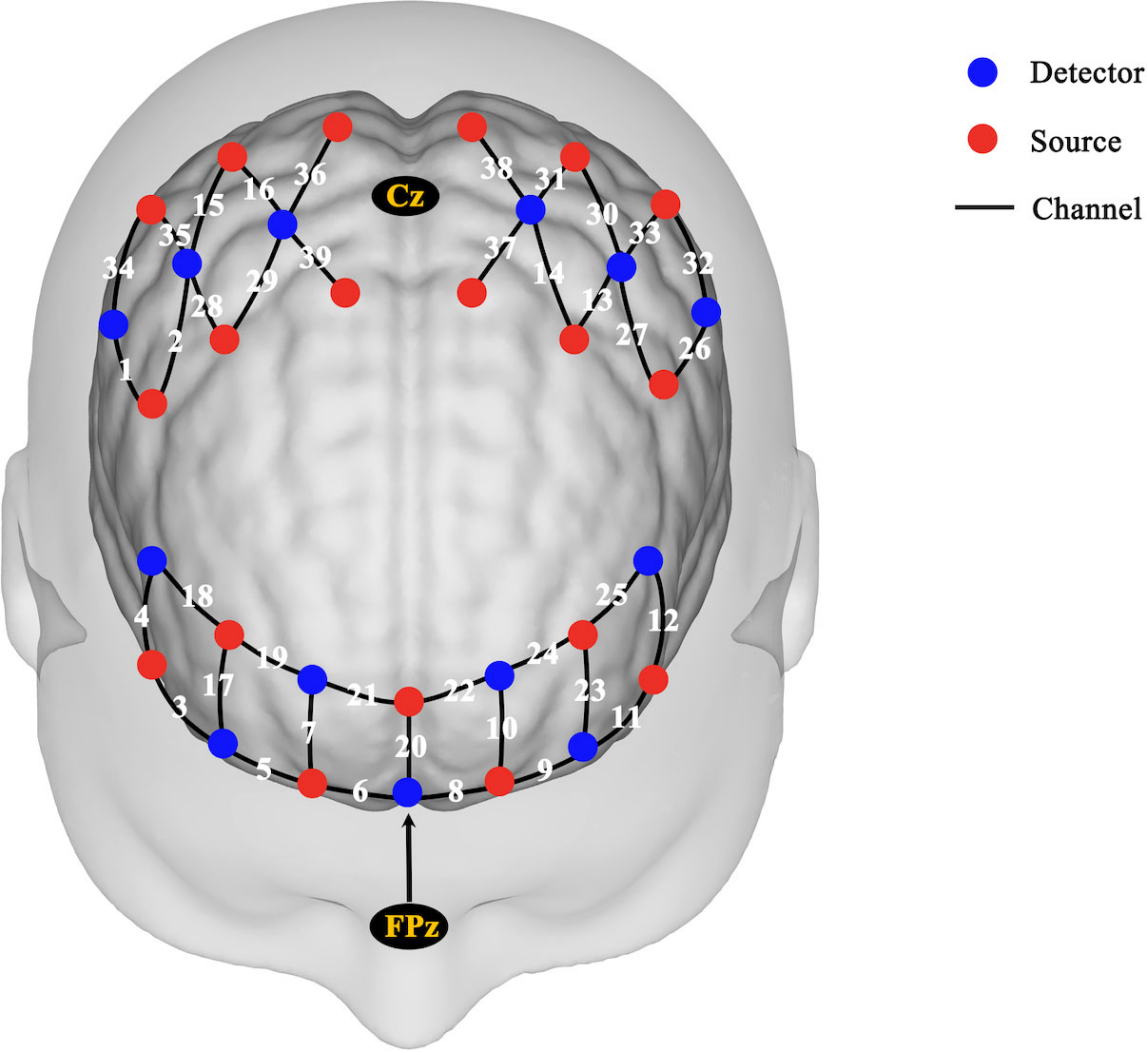
Schematic diagram of optode arrangement on the fNIRS cap.

During the static balance and TUG tasks, cortical HbO concentrations were recorded using a portable fNIRS system (NirSmart-3000A, China). The system consisted of 19 near-infrared light sources and 13 detectors, with wavelengths of 730 and 850 nm and a sampling rate of 11 Hz. Based on the role of the prefrontal and parietal cortices in cognitive–motor regulation, somatosensory processing, and balance control, and in accordance with previous fNIRS studies, these cortical regions were selected as predefined ROIs (Xu et al., 2024b; Gozdas et al., 2024). The optodes were positioned according to the international 10–20 system, forming 39 measurement channels. Sources and detectors were arranged alternately, with an average source–detector distance of 3 cm and a range of 2.7–3.3 cm (**Figure 3**).

In addition, the spatial coordinates of all measurement channels were recorded using a three-dimensional digitizer (NirSpace, China). The cortical correspondence of each channel was determined according to Brodmann areas using MRIcro software (Chris Rorden) (**Table 2**). A total of 14 ROIs were included in the analysis, with seven ROIs in each hemisphere.

**Table 2:** Corresponding brain regions for each channel.

Cortex	Regions of interest	Channel
Left prefrontal cortex	Dorsolateral prefrontal cortex	23, 24, 11
	Inferior frontal gyrus, triangular part	12, 25
	Frontal pole	10, 20, 22, 9
	Orbitofrontal cortex	8
Left parietal cortex	Primary somatosensory cortex	30, 32, 33
	Primary motor cortex	31, 38
	Premotor and supplementary motor cortex	13, 14, 27, 37, 26
Right prefrontal cortex	Dorsolateral prefrontal cortex	3, 17, 19
	Inferior frontal gyrus, triangular part	4, 18
	Frontal pole	5, 7, 21
	Orbitofrontal cortex	6
Right parietal cortex	Primary somatosensory cortex	34, 15, 35
	Primary motor cortex	16, 36
	Premotor and supplementary motor cortex	1, 2, 28, 29, 39

### 2.4 Outcome measures

#### 2.4.1 Static balance outcomes

Static balance was assessed using COP-derived parameters. The COP signals were filtered using a Butterworth low-pass filter with a cutoff frequency of 10 Hz (Hijmans et al., 2009). The following COP-based variables were then calculated: root mean square displacement (RMS), medio–lateral root mean square displacement (ML-RMS), anterior–posterior root mean square displacement (AP-RMS), mean COP velocity (V), medio–lateral mean velocity (ML-V), anterior–posterior mean velocity (AP-V), and sway area (AREA) (Kodesh et al., 2021). Lower values of these parameters indicate better postural control and greater postural stability.

#### 2.4.2 Dynamic balance outcomes

TUG: The primary outcome of the TUG was task completion time. The mean completion time across three trials was used for statistical analysis. A shorter completion time indicated better dynamic balance and functional mobility.

POMA: The POMA was used as a supplementary measure to assess functional balance performance during postural adjustment and gait tasks (Tinetti, 1986). The scale consists of a balance subscale ranging from 0 to 16 points and a gait subscale ranging from 0 to 12 points, with a maximum total score of 28 points. Each item was scored according to the participant’s observed performance during postural adjustment and walking. Higher scores indicated better balance and gait function and a lower risk of falls.

#### 2.4.3 Cortical HbO outcomes

In fNIRS studies (Pinti et al., 2020; Ferrari and Quaresima, 2012; Herold et al., 2018), HbO signals are commonly used as the primary indicator of task-related cortical hemodynamic responses because they generally show larger amplitude changes, higher signal-to-noise ratio, and greater sensitivity to regional cerebral blood flow responses than deoxygenated hemoglobin (HbR) signals. HbO responses have also been widely used in previous fNIRS studies examining postural control and walking-related cortical hemodynamic responses (Maidan et al., 2016b). Therefore, changes in HbO concentration were selected as the primary neurophysiological outcome in the present study. HbR data were preprocessed together with HbO signals using the same pipeline, but HbO was prioritized for statistical analysis and interpretation to maintain consistency with previous balance- and gait-related fNIRS studies.

### 2.5 Data processing and statistical analysis

#### 2.5.1 fNIRS data preprocessing

fNIRS data preprocessing was performed using the Homer2 toolbox in MATLAB R2021a. Raw light intensity signals were first converted into optical density (OD) signals. Motion artifacts were detected using an amplitude-based thresholding approach. Specifically, signal segments showing changes exceeding 10 times the standard deviation within a 0.5-s moving window were identified as motion-contaminated segments and excluded from subsequent analysis. The remaining OD signals were then band-pass filtered at 0.01–0.1 Hz to reduce physiological noise, including respiration, cardiac activity, and Mayer waves (Maidan et al., 2016a). The filtered OD signals were subsequently converted into changes in HbO and HbR concentrations using the modified Beer–Lambert law. Baseline correction was performed to reduce the influence of systematic drift, with the mean value during the final 5 s of the resting phase used as the baseline. Short-separation channels were not included in the present fNIRS montage; therefore, no specific regression of superficial extracerebral hemodynamic signals was performed. Accordingly, the processed HbO signals were interpreted as task-related cortical hemodynamic responses after standard preprocessing.

#### 2.5.2 Statistical analysis

All statistical analyses were performed using IBM SPSS Statistics version 26.0. Normality and homogeneity of variance were assessed using the Shapiro–Wilk and Levene’s tests, respectively. Continuous variables were compared among groups using one-way ANOVA, with Kruskal–Wallis tests considered for variables violating ANOVA assumptions. Categorical variables were analyzed using the chi-square test. When a significant group effect was observed, Bonferroni-corrected *post hoc* comparisons were performed. To reduce the risk of type I error from repeated testing, FDR correction was applied to omnibus p values within predefined outcome domains. For behavioral outcomes, FDR correction was performed separately for static balance variables under the EO and EC conditions and for dynamic balance variables. For HbO outcomes, FDR correction was applied across ROIs within each task condition. FDR-adjusted p values were reported where appropriate, with statistical significance set at adjusted p < 0.05. Partial eta squared (ηp²) was reported as the effect size.

## 3 Results

### 3.1 Static balance control across different habitual exercise modalities

#### 3.1.1 Behavioral outcomes of static balance control

One-way ANOVA revealed significant group differences in COP-derived parameters under the EO condition, including AREA (*F*_(2, 54)_ = 24.679, *p <* 0.001, *ηp² =* 0.492), ML-V (*F*_(2, 54)_ *=* 9.765, *p <* 0.001, *ηp² =* 0.277), AP-V (*F*_(2, 54)_ = 4.770, *p =* 0.013, *ηp² =* 0.158), ML-RMS (*F*_(2, 54)_ *=* 16.669, *p <* 0.001, *ηp² =* 0.395), AP-RMS (*F*_(2, 54)_ = 3.363, *p =* 0.042, *ηp²* = 0.117), and RMS (*F*_(2, 54)_ *=* 6.160, *p =* 0.004, *ηp² =* 0.195) (**Table 3**). Under the EC condition, significant group differences were observed in AREA (*F*_(2, 54)_ *=* 12.791, *p <* 0.001, *ηp² =* 0.334), ML-V (*F*_(2, 54)_ *=* 7.276, *p =* 0.002, *ηp² =* 0.222), and ML-RMS (*F*_(2, 54)_ = 13.050, *p <* 0.001, *ηp² =* 0.339).

**Table 3:** Static balance outcomes across different exercise modalities.

Indicator	Condition	Tai Chi group	Brisk walking group	Control group	*F*	*p*	*ηp²*
AREA (mm²)	EO	926.14 ± 329.56	1277.05 ± 424.96	2104.15 ± 715.02	24.679	<0.001	0.492
	EC	1411.54 ± 717.52	1727.79 ± 544.24	3039.25 ± 1527.62	12.791	<0.001	0.334
ML-V (mm/s)	EO	4.13 ± 0.92	5.10 ± 1.15	6.73 ± 2.72	9.765	<0.001	0.277
	EC	4.90 ± 1.46	5.83 ± 1.82	6.82 ± 1.18	7.276	0.002	0.222
AP-V (mm/s)	EO	8.39 ± 1.52	9.38 ± 2.07	10.58 ± 2.63	4.770	0.013	0.158
	EC	10.68 ± 3.12	11.06 ± 2.87	12.11 ± 2.64	1.179	0.316	0.044
V (mm/s)	EO	10.14 ± 1.74	13.08 ± 6.97	13.80 ± 3.94	3.032	0.057	0.106
	EC	12.71 ± 3.31	15.65 ± 8.32	15.20 ± 2.59	1.557	0.221	0.058
ML-RMS (mm)	EO	2.45 ± 0.65	3.33 ± 1.09	4.20 ± 0.93	16.669	<0.001	0.395
	EC	3.20 ± 1.26	3.31 ± 1.03	5.13 ± 1.49	13.050	<0.001	0.339
AP-RMS (mm)	EO	5.02 ± 1.16	5.64 ± 1.58	6.28 ± 1.61	3.363	0.042	0.117
	EC	5.67 ± 1.37	6.00 ± 1.64	6.75 ± 2.39	1.603	0.211	0.059
RMS (mm)	EO	5.66 ± 1.16	7.04 ± 2.29	7.66 ± 1.62	6.160	0.004	0.195
	EC	6.64 ± 1.57	7.56 ± 2.49	8.57 ± 2.71	3.145	0.052	0.110

AREA, sway area; V, mean velocity; RMS, root mean square displacement; AP, anterior–posterior direction; ML, medio–lateral direction; EO, eyes open; EC, eyes closed. The reported p values are FDR-adjusted p values.

**Figure 4:**
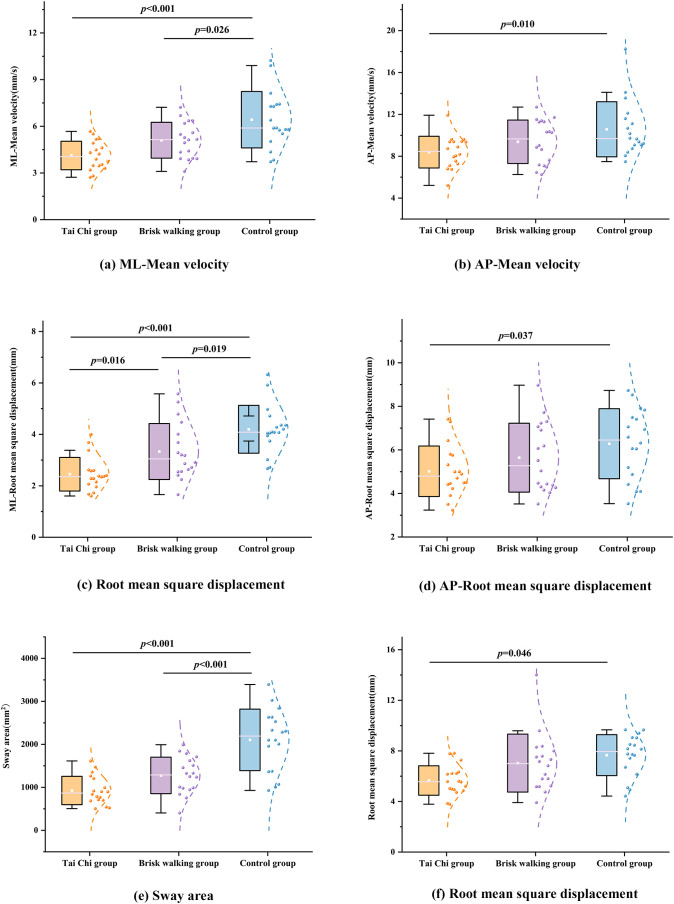
Static balance outcomes under the eyes-open condition among the three groups. **(a)** ML-mean velocity; **(b)** AP-mean velocity; **(c)** ML-root mean square displacement; **(d)** AP-root mean square displacement; **(e)** sway area; **(f)** root mean square displacement.

**Figure 5:**
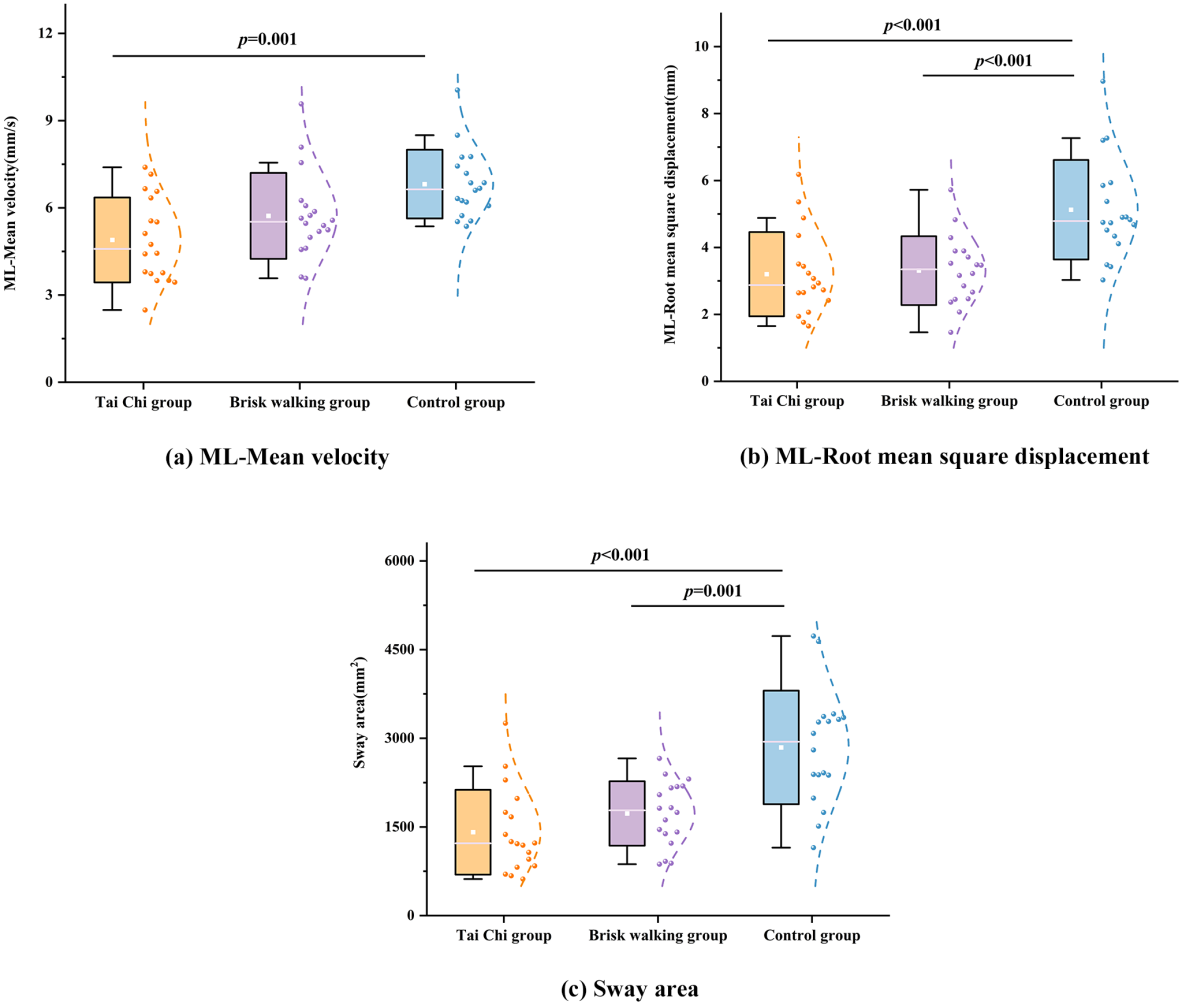
Static balance outcomes under the eyes-closed condition among the three groups. **(a)** ML-mean velocity; **(b)** ML-root mean square displacement; **(c)** sway area.

*Post hoc* multiple comparisons (**Figure 4**, **5**) showed that, compared with the control group, the brisk walking group exhibited significantly lower values in AREA (EO: *p <* 0.001; EC: *p =* 0.001), ML-V (EO: *p =* 0.026), and ML-RMS (EO: *p =* 0.019; EC: *p <* 0.001). Similarly, the Tai Chi group demonstrated significantly lower values than the control group in AREA (EO: *p <* 0.001; EC: *p <* 0.001), ML-V (EO: *p <* 0.001; EC: *p =* 0.001), AP-V (EO: *p =* 0.010), ML-RMS (EO: *p <* 0.001; EC: *p <* 0.001), AP-RMS (EO: *p =* 0.037), and RMS (EC: *p =* 0.046). Furthermore, compared with the brisk walking group, the Tai Chi group showed significantly lower ML-RMS under the EO condition (*p =* 0.016), indicating better medio–lateral postural stability.

#### 3.1.2 HbO concentration changes during static balance tasks

**Figure 6:**
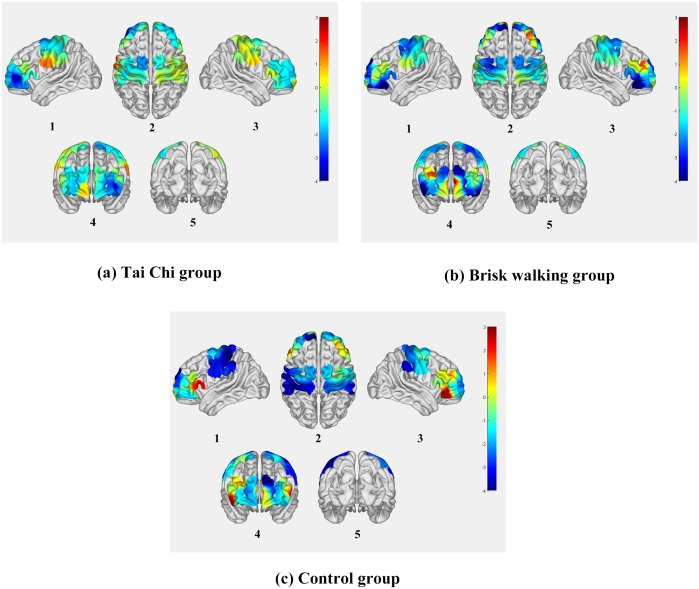
HbO concentration during eyes-open standing across the three groups (×10^-7^mmol/L). **(a)** Tai Chi group; **(b)** Brisk walking group; **(c)** Control group. The color scale represents changes in HbO concentration, with warmer colors indicating higher HbO responses and cooler colors indicating lower HbO responses. Brain views are arranged as follows: 1, left view; 2, top view; 3, right view; 4, front view; and 5, back view.

**Figure 7:**
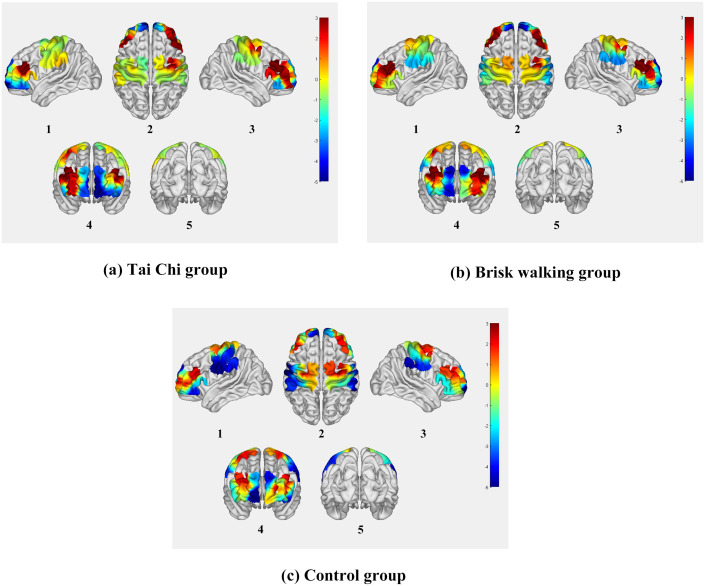
HbO concentration during eyes-closed standing across the three groups (×10^-7^ mmol/L). **(a)** Tai Chi group; **(b)** Brisk walking group; **(c)** Control group. The color scale represents changes in HbO concentration, with warmer colors indicating higher HbO responses and cooler colors indicating lower HbO responses. Brain views are arranged as follows: 1, left view; 2, top view; 3, right view; 4, front view; and 5, back view.

One-way ANOVA revealed significant group differences in HbO concentrations in the left primary somatosensory cortex (L-S1) under both EO conditions (*F*(2, 54) *=* 3.537, *p =* 0.037, *ηp² =* 0.122) and EC conditions (*F*(2, 54) *=* 4.783, *p =* 0.012, *ηp² =* 0.158) (**Table 4**). *Post hoc* multiple comparisons (**Figure 6**, **7**) showed that the Tai Chi group exhibited significantly higher HbO concentrations than the control group under both EO conditions (*p =* 0.047) and EC conditions (*p =* 0.011). In contrast, no significant differences were observed between the brisk walking group and the control group under either condition.

**Table 4:** Cortical HbO responses during static balance tasks across groups (×10^-7^ mmol/L).

Indicator	Condition	Tai Chi group	Brisk walking group	Control group	*F*	*p*	*ηp²*
L-OFC	EO	-1.27 ± 10.51	-0.02 ± 11.16	0.05 ± 6.75	0.107	0.899	0.004
	EC	-5.38 ± 7.24	-1.03 ± 10.55	0.28 ± 7.92	2.095	0.133	0.076
R-OFC	EO	0.58 ± 5.24	0.04 ± 8.77	-1.83 ± 4.21	0.712	0.495	0.027
	EC	-3.50 ± 6.89	-4.38 ± 10.99	-6.69 ± 8.11	0.625	0.539	0.024
L-DLPFC	EO	-2.69 ± 3.46	-1.58 ± 3.93	-1.44 ± 5.42	0.443	0.644	0.017
	EC	-1.14 ± 6.39	1.16 ± 7.43	-1.00 ± 7.72	0.576	0.566	0.022
R-DLPFC	EO	-1.04 ± 3.43	-1.64 ± 7.56	0.16 ± 4.06	0.532	0.591	0.020
	EC	1.31 ± 5.63	-1.64 ± 8.48	1.66 ± 7.64	1.094	0.343	0.041
L-IFG	EO	-0.65 ± 3.19	-1.29 ± 3.89	1.01 ± 9.71	0.637	0.533	0.024
	EC	1.97 ± 4.55	2.54 ± 6.35	-0.02 ± 5.69	1.047	0.358	0.039
R-IFG	EO	-0.74 ± 5.91	-1.09 ± 9.87	-0.13 ± 3.08	0.091	0.913	0.004
	EC	4.16 ± 11.37	3.87 ± 8.32	-0.18 ± 7.10	1.276	0.288	0.048
L-FP	EO	-1.38 ± 4.66	-1.68 ± 6.10	-1.85 ± 4.57	0.039	0.962	0.002
	EC	-3.57 ± 5.78	-0.46 ± 6.69	-1.72 ± 5.56	1.209	0.307	0.045
R-FP	EO	-1.39 ± 3.17	-0.91 ± 3.02	-1.03 ± 3.66	0.107	0.898	0.004
	EC	-1.46 ± 4.73	-2.09 ± 8.01	-1.31 ± 5.50	0.078	0.925	0.003
L-S1	EO	-0.70 ± 2.99	-1.21 ± 3.40	-3.45 ± 3.50	3.533	0.037	0.122
	EC	-0.18 ± 3.59	-1.41 ± 3.20	-3.88 ± 4.12	4.783	0.012	0.158
R-S1	EO	-0.03 ± 3.68	-1.36 ± 4.78	-2.75 ± 3.03	2.192	0.122	0.079
	EC	-0.52 ± 3.34	-1.32 ± 3.58	-2.65 ± 3.81	1.610	0.210	0.059
L-M1	EO	-0.68 ± 3.96	-1.09 ± 4.96	-3.34 ± 2.73	2.324	0.108	0.084
	EC	-0.60 ± 3.46	-0.09 ± 6.08	-1.12 ± 5.75	0.175	0.840	0.007
R-M1	EO	-0.35 ± 4.24	-0.34 ± 5.86	-2.37 ± 3.39	1.161	0.321	0.044
	EC	-0.81 ± 3.67	0.06 ± 6.63	-1.08 ± 6.12	0.203	0.817	0.008
L-PMC/SMA	EO	-0.95 ± 3.09	-1.97 ± 4.26	-2.29 ± 2.64	0.764	0.471	0.029
	EC	-0.45 ± 3.30	-0.42 ± 4.60	-1.37 ± 3.90	0.334	0.718	0.013
R-PMC/SMA	EO	-0.39 ± 2.99	-1.85 ± 4.24	-1.04 ± 2.80	0.830	0.442	0.032
	EC	0.79 ± 3.04	0.13 ± 4.00	0.47 ± 4.96	0.117	0.890	0.005

OFC, orbitofrontal cortex; DLPFC, dorsolateral prefrontal cortex; IFG, triangular part of the inferior frontal gyrus; FP, frontal pole; S1, primary somatosensory cortex; M1, primary motor cortex; PMC/SMA, premotor cortex and supplementary motor area; L, left hemisphere; R, right hemisphere; EO, eyes open; EC, eyes closed. The reported p values are FDR-adjusted p values.

### 3.2 Dynamic balance control across different habitual exercise modalities

#### 3.2.1 Behavioral outcomes of dynamic balance control

**Figure 8:**
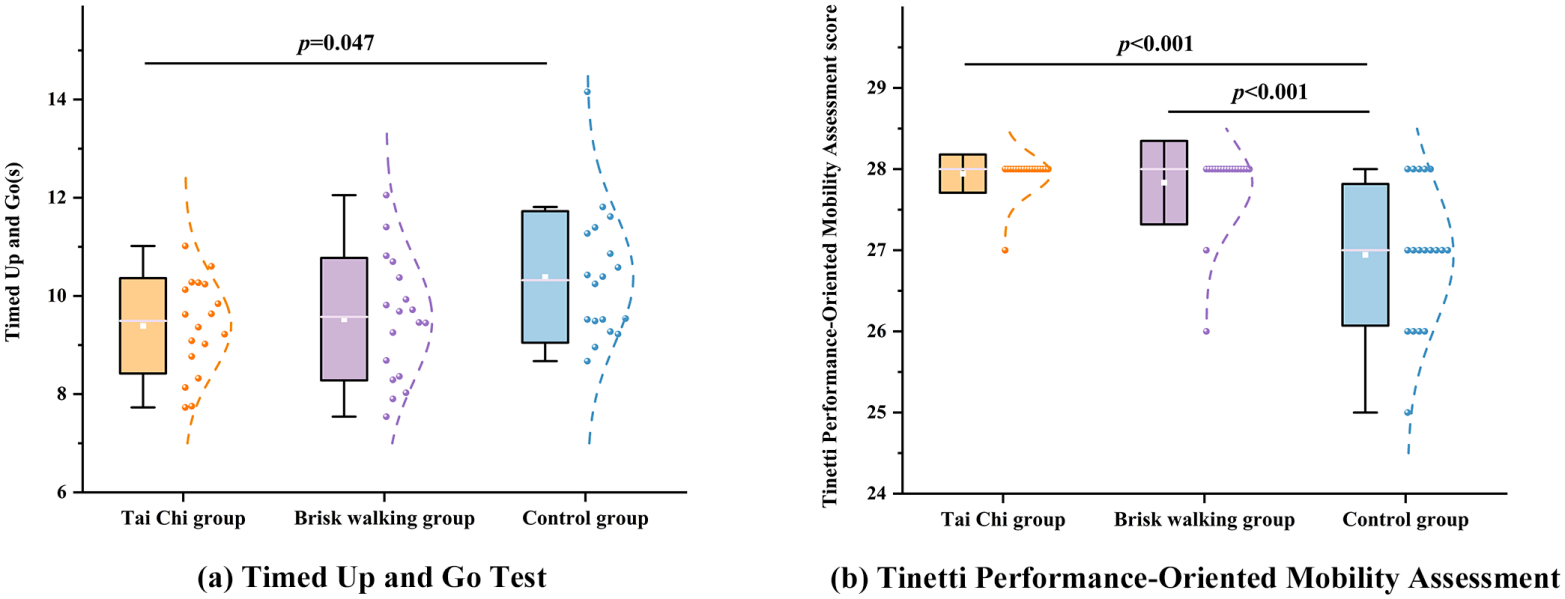
Dynamic balance outcomes across the three groups. **(a)** Timed Up and Go Test; **(b)** Tinetti Performance-Oriented Mobility Assessment.

One-way ANOVA (**Table 5**) showed significant group differences in both TUG performance (*F*_(2, 54)_ *=* 3.667, *p =* 0.033, *ηp² =* 0.126) and POMA scores (*F*_(2, 54)_ *=* 14.997, *p <* 0.001, *ηp² =* 0.370) among the three groups. *Post hoc* pairwise comparisons (**Figure 8**) showed that both the brisk walking group and the Tai Chi group had significantly higher POMA scores than the control group (*p <* 0.001). In addition, the Tai Chi group showed significantly shorter TUG completion time than the control group (*p* = 0.047), indicating better dynamic balance and functional mobility. POMA findings were interpreted as supplementary evidence of functional balance performance.

**Table 5:** Dynamic balance outcomes across different exercise modalities.

Indicator	Tai Chi group	Brisk walking group	Control group	*F*	*p*	*ηp²*
POMA	27.94 ± 0.24	27.83 ± 0.51	26.94 ± 0.87	14.997	<0.001	0.370
TUG (s)	9.39 ± 0.97	9.53 ± 1.25	10.39 ± 1.34	3.667	0.033	0.126

TUG, Timed Up and Go test; POMA, total score of the Tinetti Performance-Oriented Mobility Assessment.

#### 3.2.2 HbO concentration changes during the TUG task

**Figure 9:**
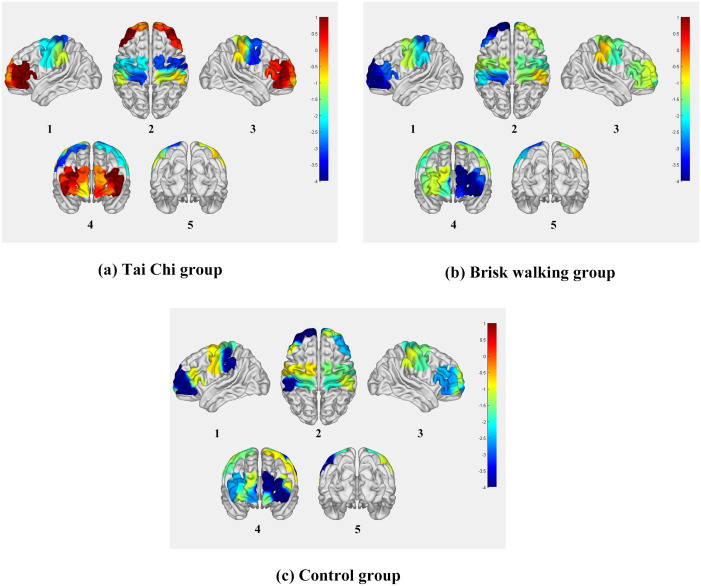
HbO concentration during walking across the three groups (×10^-7^ mmol/L). **(a)** Tai Chi group; **(b)** Brisk walking group; **(c)** Control group. The color scale represents changes in HbO concentration, with warmer colors indicating higher HbO responses and cooler colors indicating lower HbO responses. Brain views are arranged as follows: 1, left view; 2, top view; 3, right view; 4, front view; and 5, back view.

One-way ANOVA revealed a significant group difference in HbO concentrations in the right dorsolateral prefrontal cortex (R-DLPFC) during walking (*F*(2, 54) *=* 3.409, *p =* 0.041, *ηp² =* 0.118) (**Table 6**). *Post hoc* multiple comparisons (**Figure 9**) showed that the Tai Chi group exhibited significantly higher HbO concentrations than the control group during walking (*p =* 0.037). In contrast, no significant difference was observed between the brisk walking group and the control group.

**Table 6:** Changes in HbO concentration during walking across different exercise modalities (×10^-7^ mmol/L).

Indicator	Tai Chi group	Brisk walking group	Control group	*F*	*p*	*ηp²*
L-OFC	0.30 ± 5.96	-3.21 ± 11.41	-1.50 ± 5.24	0.862	0.428	0.033
R-OFC	-0.85 ± 2.75	-1.77 ± 5.07	-2.70 ± 5.15	0.769	0.469	0.029
L-DLPFC	1.28 ± 6.27	-3.90 ± 11.88	-4.67 ± 11.69	1.782	0.179	0.065
R-DLPFC	0.95 ± 2.32	-1.23 ± 3.43	-2.66 ± 5.93	3.409	0.041	0.118
L-IFG	2.67 ± 14.80	-2.92 ± 10.35	-1.03 ± 4.49	1.258	0.293	0.047
R-IFG	0.17 ± 8.39	-1.52 ± 4.86	-2.53 ± 5.04	0.843	0.436	0.032
L-FP	-0.36 ± 9.18	-5.04 ± 16.18	-4.83 ± 11.75	0.777	0.465	0.030
R-FP	0.01 ± 5.08	-1.07 ± 5.20	-1.18 ± 10.48	0.141	0.869	0.005
L-S1	-1.21 ± 4.30	-2.30 ± 3.27	-3.93 ± 6.64	1.381	0.260	0.051
R-S1	-0.69 ± 3.41	-0.59 ± 3.48	-1.05 ± 4.37	0.073	0.929	0.003
L-M1	-3.16 ± 10.28	-3.11 ± 6.12	-1.82 ± 4.98	0.185	0.831	0.007
R-M1	-1.18 ± 9.01	-1.45 ± 3.75	-1.96 ± 4.25	0.076	0.927	0.003
L-PMC/SMA	-2.17 ± 5.22	-1.31 ± 3.71	-0.83 ± 6.32	0.305	0.739	0.012
R-PMC/SMA	-3.13 ± 5.43	-1.71 ± 3.98	-1.60 ± 4.01	0.416	0.662	0.016

OFC, orbitofrontal cortex; DLPFC, dorsolateral prefrontal cortex; IFG, triangular part of the inferior frontal gyrus; FP, frontal pole; S1, primary somatosensory cortex; M1, primary motor cortex; PMC/SMA, premotor cortex and supplementary motor area; L, left hemisphere; R, right hemisphere. The reported p values are FDR-adjusted p values.

## 4 Discussion

The present study compared balance performance and cortical hemodynamic responses among the Tai Chi group, brisk walking group, and non-exercise control group to examine how different exercise modalities are associated with balance control and task-related cortical hemodynamic response patterns in older adults with MCI. The results showed that, compared with the control group, both the Tai Chi and brisk walking groups exhibited better balance performance to varying extents. Notably, the Tai Chi group showed more comprehensive advantages in balance-related outcomes, accompanied by higher HbO responses in task-related ROIs during specific balance tasks. These findings suggest that habitual exercise patterns are associated with balance performance and task-related cortical hemodynamic responses in older adults with MCI, with Tai Chi showing more favorable associations than brisk walking. However, because participants were grouped according to their habitual exercise behaviors rather than randomized allocation, these associations may have been influenced by residual confounding and healthy exerciser bias, including potential differences in lifestyle, baseline mobility, cognitive engagement, social interaction, and physical fitness.

Balance control is not a single motor outcome but a complex process involving postural maintenance, center-of-mass regulation, gait control, and movement transitions (Horak, 2006). Based on this multidimensional nature, the present study further examined the association of different exercise modalities with balance performance in older adults with MCI from both static and dynamic perspectives. The static balance results showed that both the Tai Chi and brisk walking groups performed better than the control group across multiple postural sway parameters, suggesting that regular exercise may be associated with reduced postural sway during quiet standing and a greater ability to maintain body stability under relatively stable conditions (Wang et al., 2024; Bai et al., 2021; Wu, 2002).

The dynamic balance results further showed that the Tai Chi group performed better than the control group in TUG, the primary dynamic balance outcome, while POMA provided supplementary evidence of functional balance and gait performance. Because POMA scores were close to the maximum value across groups, the POMA findings should be interpreted conservatively and considered together with TUG performance. Together, these findings suggest that habitual exercise patterns may be associated with both static postural control and dynamic balance regulation in older adults with MCI (Latorre-Román et al., 2026; Wang et al., 2024).

Compared with the brisk walking group, the Tai Chi group showed more pronounced advantages in both static and dynamic balance performance. The more favorable balance performance observed in the Tai Chi group may be related to the task-specific characteristics of Tai Chi practice. Brisk walking, as a rhythmic aerobic exercise, may be associated with better balance performance through walking endurance, lower-limb function, and basic mobility capacity (Latorre-Román et al., 2026; Paillard et al., 2004). In contrast, Tai Chi involves a series of complex motor patterns, including slow and controlled weight shifting, transitions between weighted and unweighted stances, single-leg support, trunk rotation, and coordinated movements of the upper and lower limbs (Law and Li, 2022). During Tai Chi practice, individuals are required to continuously monitor body posture, regulate the position of the center of mass, and actively adjust their movements in response to changing task demands (Shao et al., 2022; Huang et al., 2022; Zhang et al., 2021). These training characteristics are closely related to key components of balance control, such as anticipatory postural adjustment, weight transfer, and motor coordination (Huang et al., 2022). These task-specific features may partly explain why habitual Tai Chi practice was associated with more favorable balance performance in this study (Tsang and Hui-Chan, 2008).

In addition, the present study found that the cortical regions showing significant HbO differences were not consistent between static and dynamic balance tasks. During quiet standing, the Tai Chi group exhibited significantly higher HbO concentrations in the L-S1 than the control group, whereas during walking, the Tai Chi group showed significantly higher HbO concentrations in the R-DLPFC than the control group. These findings suggest that the relationship between habitual Tai Chi practice and balance performance may be task dependent (Weng et al., 2023; Dong et al., 2024). Static standing primarily requires individuals to perceive subtle body sway and rely on proprioceptive feedback for fine postural adjustments, thereby preferentially involving the S1 region, which is closely associated with somatosensory processing (Gerber et al., 2024; Smith et al., 2023). In contrast, dynamic balance tasks involve gait initiation, walking, turning, and movement transitions, which place greater demands on attentional allocation, motor planning, and spatial monitoring and may therefore rely more strongly on prefrontal regulation, particularly the involvement of the DLPFC (Holzter et al., 2024; Chen et al., 2022b). In other words, static balance emphasizes postural maintenance based on sensory feedback, whereas dynamic balance places greater emphasis on cognitively mediated motor control. This task-specific pattern of cortical involvement may therefore reflect differences in the neural regulatory demands imposed by different balance tasks.

It should be noted that higher HbO responses should not be interpreted solely as improved neural efficiency. In older adults and individuals with MCI, higher task-related HbO responses may also reflect the recruitment of additional neural resources or compensatory neural engagement required to maintain task performance (Xu et al., 2024a). According to the compensatory neural scaffolding theory, older adults may recruit additional neural resources to support behavioral performance when facing increased cognitive or motor-control demands (Fan et al., 2025). Therefore, the higher HbO responses observed in the L-S1 and R-DLPFC in the Tai Chi group may reflect greater task-related cortical engagement during static and dynamic balance tasks, rather than simply indicating more efficient neural processing. From the perspective of task characteristics, habitual Tai Chi practice may be associated with static balance performance by facilitating somatosensory information processing and supporting the perception of changes in body position (Chen et al., 2023; Cui et al., 2024). During dynamic balance tasks, Tai Chi practice may be related to greater involvement of prefrontal-mediated cognitive–motor regulation, thereby supporting postural stability during walking and movement transitions (Chen et al., 2025; Dong et al., 2025). In contrast, although the brisk walking group demonstrated better performance than the control group in several behavioral balance measures, no significant cortical HbO differences were observed. This finding may suggest that the balance-related advantages associated with brisk walking are more closely related to physical factors, such as walking endurance, lower-limb function, general mobility, or cardiorespiratory fitness, rather than to detectable cortical hemodynamic changes within the predefined brain regions examined in the present study (Weng et al., 2023). Taken together, these findings suggest that different exercise modalities may correspond to distinct balance regulation characteristics: Tai Chi may be more strongly associated with task-related cortical involvement across multiple domains, including sensory feedback, postural control, and cognitive–motor integration, whereas brisk walking may primarily reflect adaptations at the level of physical function.

The present study provides preliminary behavioral and neurophysiological evidence for the association between habitual exercise modalities and balance control in older adults with MCI. Nevertheless, several limitations should be acknowledged. First, the relatively small and predominantly female sample may limit statistical power and generalizability, particularly given the multiple behavioral outcomes and fNIRS ROIs analyzed. Potential sex-related differences in balance control or cortical hemodynamic responses may further affect the generalizability of the findings. In addition, because short-separation channels were not included, the HbO findings may have been influenced by residual extracerebral hemodynamic signals; therefore, the cortical HbO findings should be interpreted as preliminary and exploratory. Second, although POMA was used as a supplementary measure of dynamic balance, its near-ceiling scores may have reduced sensitivity to subtle between-group differences; therefore, these results should be interpreted together with TUG performance. Finally, because this was a cross-sectional observational study based on self-reported habitual exercise patterns, causal relationships cannot be inferred. In addition, exercise intensity, adherence, and overall physical activity were not objectively monitored, and potential self-selection bias related to lifestyle, motivation, cognitive engagement, or health behavior cannot be fully excluded. Future longitudinal randomized studies with larger and more balanced samples and objective exercise monitoring are needed to confirm these findings.

In summary, both brisk walking and Tai Chi were associated with better static and dynamic balance performance to varying extents, with Tai Chi showing more favorable associations. As an exercise modality that integrates physical training with cognitive engagement, Tai Chi may be a promising candidate for future balance-oriented intervention studies in older adults with MCI.

## 5 Conclusion

This study suggests that different habitual exercise modalities are associated with balance control in older adults with MCI. Compared with brisk walking and no regular structured exercise, habitual Tai Chi practice showed more favorable associations with both behavioral balance performance and task-related cortical hemodynamic responses. These findings suggest that Tai Chi may be a promising exercise modality for future balance-oriented intervention studies and fall-risk prevention strategies in older adults with MCI. However, longitudinal randomized controlled trials are needed to determine whether Tai Chi can produce causal changes in balance function and task-related cortical hemodynamic responses in this population.

## Data Availability

The original contributions presented in the study are included in the article/supplementary material. Further inquiries can be directed to the corresponding author.
